# Tritium-Labeled Compounds I. Radioassay of Tritium-Labeled Compounds in “Infinitely Thick” Films With a Windowless, Gas-Flow, Proportional Counter^1^

**DOI:** 10.6028/jres.063A.011

**Published:** 1959-10-01

**Authors:** Horace S. Isbell, Harriet L. Frush, Ruth A. Peterson

## Abstract

A simple, sensitive, and reliable technique has been devised for the radioassay of nonvolatile, water-soluble tritium compounds. The substance to be analyzed is dissolved in an aqueous solution of a thickening agent, preferably sodium *O*-(carboxymethyl) cellulose or sodium alginate. The solution is placed in a shallow planchet, and after evaporation of the water, the resulting film, which is “infinitely thick” to tritium *beta* particles, is counted with a 2*π*, windowless, gas-flow, proportional counter. By means of an empirical factor, determined with a substance of known radioactivity, the counts are converted to microcuries. In a film having a thickness of 0.7 mg/cm^2^, the counting efficiency is about 4 percent; the standard deviation from the mean, obtained in a series of routine measurements, was less than 2 percent. An assay can readily be made with tritium-containing material having 0.01 microcurie of radioactivity. The method, which is applicable to nonvolatile, water-soluble solids, solutions, or liquids, is suitable for routine analyses.

## 1. Discussion

Tritium is one of the cheapest and most versatile radioisotopes for use as a tracer in chemical reactions. However, its widespread use has been hampered by a lack of convenient methods of analysis. In connection with the development of methods for the preparation of position-labeled, radioactive carbohydrates containing tritium, a simple, rapid technique was needed for the assay of radioactivity of nonvolatile, tritium-labeled materials. Liquid scintillation counters [1],^2^ although very satisfactory for assaying dissolved samples, are both expensive and unavailable to many workers. The conversion of materials to gases, for counting in ionization chambers [1] is laborious for routine analyses. Several workers have counted tritium-labeled materials from “infinitely thick” films with proportional counters [2 to 7]. A tritium-containing solid, if available in relatively large quantity, may be packed into a cupped planchet and counted [2, 3, 4]; this low-efficiency method has chiefly been used for comparisons, rather than for the determination of absolute activities. Alternatively, an infinitely thick film of a nonvolatile liquid has been employed [5], or a solid film has been deposited on a planchet by evaporation of the solvent from a solution [6, 7]. The last-mentioned method is probably the most versatile, but it suffers from the fact that crystallization on the planchet can render the surface area of the film uncertain and variable [5, 7], and may even cause contamination of the counter during flushing [6].

For routine radioassay of nonvolatile, water-soluble, tritiated materials in a windowless, gas-flow, proportional counter, a technique has been developed in this laboratory that avoids difficulty caused by crystallization and permits the counting, from uniform, infinitely thick films, of materials available in small quantity. The radioactive substance is dis-solved in an aqueous solution of a thickening agent, preferably sodium *O*-(carboxymethyl) cellulose or sodium alginate.^3^ The amount of sample and thickening solution are so adjusted that the thickness of the film formed on the planchet after evaporation of the water is more than the maximum range of the tritium *beta* particles, (infinitely thick, approximately 0.7 mg/cm^2^ for the material used [9]). The films adhere well to the planchets, and the thickener prevents crystallization. The counts are converted to micro-curies (*μ*c) by means of an empirical factor determined under the same conditions with a sample of known radioactivity.^4^ Although the counting efficiency of a film having a thickness of 0.7 mg/cm^2^ is only about 4 percent, the reproducibility of the method corresponds to a standard deviation of less than 2 percent. A satisfactory determination can be made with as little as 0.01 *μ*c of a tritium-labeled material on a 2-in. planchet. Because of its simplicity the method is suitable for routine analyses. The accuracy of the method depends on the absolute activity of the material used as standard. For the work reported here, d-glucos*e-1-t* was standardized by comparing it with the NBS tritium oxide standard.

In the course of the work, a study was made with films of less than infinite thickness. Although high counting efficiencies can be obtained, uncertainty arising from uneven distribution of the sample makes counting from thin films less satisfactory than counting from infinitely thick films by the method described.

## 2. Materials and Apparatus

### 2.1. Tritium-Labeled Materials

d-Glucose-1-*t* was prepared by sodium amalgam reduction of d-glucono-*δ*-lactone in tritiated water [12] in the presence of sodium acid oxalate [13]. The sample used as a tritium standard was assayed by comparing its activity in a phosphoric acid-phosphoric anhydride solution with that of the NBS standard tritium oxide sample No. 4926; the measurements were made with a proportional counter [14]. d-Mannitol-1-*t* was prepared by reducing d-mannose with tritiated lithium borohydride [14].

### 2.2. Solution of Sodium *O*-(Carboxymethyl)cellulose

The thickening agent was commercial, medium-viscosity sodium *O*-(carboxymethyl) cellulose, CMC-12MP, a product of Hercules Powder Company, Wilmington, Del. The CMC stock solution ordinarily used for the radioassay of solid samples was prepared by dissolving 1 g of CMC, 0.5 g of anhydrous d-glucose, and 10 mg of eosin^5^ in sufficient water to give 100 ml of solution. The d-glucose serves as an organic “ballast” material and plasticizer. It was omitted entirely, or in part, in thickening solutions for use with large samples of weakly radioactive material. d-Glucitol has been used with equal success as ballast in place of d-glucose.

### 2.3. Solution of Sodium Alginate

The sodium alginate used was high-viscosity Algin, obtained from Kelco Company, San Diego, Calif. The stock solution was prepared from 0.5 g of Algin, 1 g of anhydrous d-glucose, 10 mg of eosin, and sufficient water to give 100 ml of solution.

### 2.4. Counting Equipment

Radioactivity measurements were made with a 2π, windowless, gas-flow, proportional counter, Model PC–3, obtained from Nuclear Measurements Corp., Indianapolis, Ind.; the slide holder of the instrument had been modified by inserting tubes for water circulation [15]. Commercial gas, consisting of 90 percent of argon and 10 percent of methane, was used in the counter. The gas was dried by successive passage through soda-lime and anhydrous calcium sulfate. The 2-in., stainless-steel, cupped, flat-bottomed, counting planchets used in this work were also obtained from Nuclear Measurements Corp.; they have an effective area of 21.4 cm^2^. Some of these planchets were modified by enclosing a 10-cm^2^ area with a circular groove beyond which the solution did not spread.

## 3. Recommended Procedure for Radioassay of Tritium-Labeled Compounds in Infinitely Thick Films

The solution of the radioactive sample in the aqueous thickening agent can be prepared by any convenient method. Ordinarily, radioactive solids are weighed, and then dissolved in a known volume of the stock thickening solution. A radioactive liquid, usually less than 0.5 ml, is mixed with a measured amount of the thickening solution. However, if the liquid is sufficiently radioactive, it is measured in a dilution pipet and diluted to a definite volume with the thickening solution. Approximately 1 ml of the solution to be assayed is transferred to a clean,^6^ 2-in. cupped planchet (or 500 microliters is transferred to the 10-cm^2^ area of the planchet), which is then placed under an infrared lamp. After the solvent has evaporated, the planchet is kept for about 1 hr in a desiccator over a saturated solution of potassium acetate in contact with the solid phase. This rather unusual procedure gives films of reproducible moisture content, and does not lower the count substantially. The sample is then counted in the proportional counter for 3 periods, with a total of at least 10,000 counts. Before each period the counter is flushed with the counting gas for 30 sec. It occasionally happens that successive counts show a marked trend, presumably caused by the accumulation of a static charge on the film. If this occurs, the sample is removed to the desiccator for a short time, and then recounted. The background count is determined with a film-covered planchet placed in the counting chamber.

The voltage for optimum precision must be determined under the conditions used. The optimum voltage for assay of solid samples with the equipment used in the present study was approximately 2,050 volts.

## 4. Discussion of the Procedure and Results

The activity of tritium in the sample is given by the relationship:

$$\mu c=\frac{\text{dps}}{37,000}=\text{cps}\times m\times k$$ (1)

where cps is the net count, *m* is the total solids in a volume of solution containing the radioactive sample, and *k* is a constant determined by the same method for a compound of known tritium content.

The value of *m* must be determined accurately, although the exact amount of solution placed on the planchet is not critical if the film formed from it is infinitely thick. Because of hydration, the value of *m* calculated from the ingredients in the formulation may differ slightly from that determined by weighing the film produced, under the conditions of the method, from a definite volume of the solution. Determination of the weight of the film in each analysis is laborious. This step can be avoided by determining the weight of the film formed on a planchet from exactly 1 ml of the *stock solution* of CMC or Algin under the conditions of the analysis. Because of the viscosity of the solution, a pipet, calibrated to contain 1 ml, is employed, and the solution adhering to the pipet is carefully rinsed out and added to the planchet. The film, obtained after evaporation of the water, is conditioned in the usual manner and weighed. Several determinations are made, and the average value is used for calculating *m*, the weight of the film containing the radioactive substance, on the assumption that *m* equals the weight of the film from the amount of stock solution used plus the weight of the radioactive sample. All nonvolatile matter in the formulation must be included in the calculation of *m.*

The value of *k* depends on the surface area, back-scattering, efficiency of the counter, geometry, and other factors; it has the dimension of weight^−1^. To obtain accurate results in the analysis of a series of compounds, *k* must be determined by assay of a standard tritium compound under the conditions used for the unknowns. With materials of similar character, the amount of radiation scattering is constant. Because scattering varies with the effective atomic number of the reflecting material, heavy elements such as barium increase internal scattering and may lead to small errors. These can be avoided by adding, to the standard, compounds of any heavy elements that may be present in the material to be assayed. Ions of certain metals, as, for example, lead, form insoluble compounds with both CMC and Algin. This undesirable effect can be overcome by adding Versene to the film-forming mixture.

Table 1 presents results obtained with two samples each of d-glucos*o-1-t* and d-mannitol-*1*-*t*. The standard deviations are based on the deviations in counting rates for the separate planchets from the mean counting rate for the group of planchets. Sample G_1_ was used as a standard, and its specific activity (0.0865 *μc/*mg) was carefully determined by the comparative method described under materials and apparatus. Use of this material as a control gave values of *k* under various conditions, i.e., for CMC and Algin films and for the 10-cm^2^ and 21.4-cm^2^ (2-in.) planchets. By use of these constants, the specific activities of G_2_, M_1_, and M_2_ were determined. The counting rates for films having thicknesses between 0.69 and 1.48 mg/cm^2^ showed no significant differences.

## 5. Assay of Tritium Compounds in Films of Less Than Infinite Thickness

**Figure 1:**
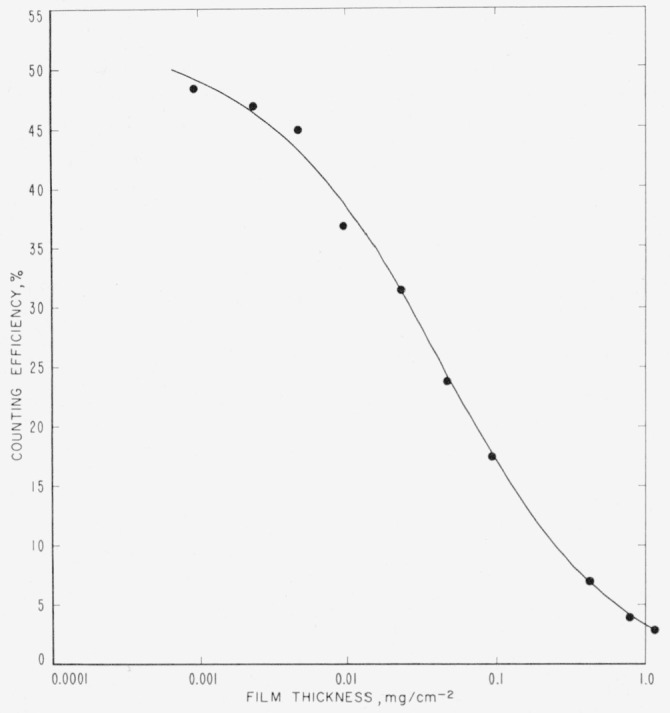
Counting efficiency as a function of film thickness.

Table 2 and figure 1 show the relative counting rate of a preparation of d-glucose-*t* as a function of source thickness. Each sample was spread on a freshly cleaned, 2-in., stainless-steel planchet, and the solvent was evaporated under an infrared lamp. The planchet was cooled in a desiccator over anhydrous calcium chloride, and then counted with the equipment described before. The first seven determinations were made on films from solutions of d-glucose-*t* in water, and the last three on films from solutions of d-glucose-*t* containing sufficient Algin to give the weight recorded. The radioactivity in thin films can be estimated by use of the data of table 2 and figure 1.

## 6. Discussion of Absolute Radioactivity

The counting rate of a tritium source in an efficient, windowless, gas-flow, proportional counter can be represented by an equation of the type [6]:

$$dps=\frac{cps}{f_{g}\times f_{b}\times f_{s}}$$ (2)

where dps is the activity of a source, and *f_g_*,*f_b_*, and *f_s_* are correction factors for the geometry of the detector, for backscattering, and for self-absorption, respectively. The geometry factor, *f_g_*, with a flat planchet is approximately 0.5. The backscattering factor, *f_b_*, is a function of the atomic weight of the backing material and depends somewhat on the character of the radiation and on the geometry of the counter [16]. But with an infinitely thick source, scattering from the backing material may be neglected because the only reflected radiation that reaches the counter originates in the source itself. With sources composed of carbon, hydrogen, and oxygen, the scattering factor is of the order of 5 percent, and can be neglected in obtaining an approximate estimate of the true activity. Prior workers have shown that the self-absorption factor, *f_s_* for the *beta* radiation is given by the relationship:

$$f_{s}=\frac{A_{T}}{A_{0}}=\frac{1-e^{-\mu T}}{\mu T},$$ (3)

where *μ* is the self-absorption coefficient and *A_T_* is the radiation from a source containing a unit weight of the radioactive material with a thickness *T; A*_0_ is the limiting value for *A_T_* as *T* approaches zero. If the weight of the source is *m* mg and the area is *a* cm^2^, $T=\frac{m}{a}$ and eq (3) can be rewritten:

$$f_{s}=\frac{A_{T}}{A_{0}}=\frac{1-e^{-\mu T}}{\frac{\mu m}{a}}.$$ (4)

When *T* is infinite with respect to *beta* particles, as in the analytical method, 1−*e*^−^^*μT*^=1, and eq (4) becomes:

$$\frac{mA_{T}}{A_{0}}=\frac{a}{\mu }.$$ (5)

When *T* aproaches zero, *f_s_* approaches 1, and from eq (2) and the definition of *A*_0_:

$$A_{0}={\left(\frac{cps}{m}\right)}_{f_{s}\to 1}=\frac{dps}{m}\times f_{g}\times f_{b}.$$ (6)

Since *mA_T_* is the cps of a source weighing *m* milligrams, eq (5), by substitution for *mA_T_* and *A*_0_ becomes:

$$\frac{a}{\mu }=\frac{cps}{\left(\frac{dps}{m}\right)\times f_{g}\times f_{b}}.$$ (7)

Assuming 2*π* geometry (*f*_0_=0.5) and no back-scattering (*f_b_*=1), values of 1/*μ* can be calculated from eq (7) for all of the measurements recorded in table 1. However, there is considerable uncertainty in the value of *a* for determinations made with the 2-in., commercial, flat-bottomed planchets, because of the curved edge. Hence, the best value of l/*μ* is that obtained from the measurements for the standardized sample *G*_1_ in the 10-cm^2^ areas. In the first experiment of table 1, *a*=10.0, cps=50.1, and (*dps*/*m*) *f_g_*= (0.435 × 0.0856 × 37,000/7.7) × 0.5. From these values there is obtained:

$$\frac{1}{\mu }=0.0560\;\text{mg}/{\text{cm}}^{2}.$$

The value of 1/*μ* can be used to obtain *T*_1/2_ (the half-value thickness of the radiation) and *R* (the range) from the relationships [9, 17] *T*_1/2_=0.693/*μ* and *R*=5/*μ.* With the above value of 1/*μ*, *T*_1/2_= 0.039 mg/cm^2^ and *R*=0.28 mg/cm^2^. This value of *R* can be compared with experimental values, reported in the literature, of 0.23 mg/cm^2^ [18], 0.5 mg/cm^2^ [19],^7^ 0.6 mg/cm^2^ [6], and with the value of 0.7 mg/cm^2^ based on an empirical energy-range relationship [9]. The variation in the results arises from experimental difficulties and from uncertainty in the absolute disintegration rate of the source.

It is of interest to note that extrapolation of the observed counting rates of figure 1 to zero thickness gave a value of approximately 50 percent of the absolute disintegration rate, based on the NBS tritium standard. With a stainless-steel planchet, under conditions somewhat comparable to those used here, Rydberg’s data [6] for thin films indicate a back-scattering factor of about 1.25. Thus the count would be expected to be approximately 62 percent of the absolute rate, with a stainless-steel planchet, 2*π* geometry, and no self-absorption. A low value might arise from unequal distribution of the material on the planchet. This factor is of no importance with thick samples, but could give rise to large errors with thin samples. Such errors make accurate assay of tritium materials in thin samples impracticable.

Assay of infinitely thick films is, however, highly satisfactory, and is recommended for routine, laboratory analysis. In eq (1), the empirical constant *k* includes corrections for the area of the source, geometry of the counter, self-absorption, backscattering, and conversion of total *dps* to microcuries. By use of eq (1) and (7), *k* may be related to *a*/*μ* thus:

$$k=\frac{\mu }{a\times f_{g}\times f_{b}\times 37,000}$$

However, because of uncertainties in the values for *a*, *f_g_*, and *f_b_*, the constant *k*, for the calculation of absolute radioactivity from observed counts, should be experimentally determined under the conditions of use.

## Figures and Tables

**Table 1:** Countinq rates of tritium compounds in “infinitely thick” films

Sample		Film				Counting Data			Specific activity^d^ of sample	k×10^5^
Substance^a^	Weight^b^ (*m′*)	Thickening agent	Area	Total solids (*m*)	Thickness	No. of planchets	Average count	Standard deviation^c^		
										
	*mq*		*cm*^2^	*mq*	*mg/cm*^2^		*cps*	%	*μc*/*m*′	
G_1_	0.435	CMC	10.0	7.7	0.77	5	50.1	1.04	(0.0856)	9.65
G_1_	.87	CMC	21.4	15.4	.72	5	108.7	0.86	(.0856)	4.45
G_1_	.415	Algin	10.0	8.1	.81	4	45.1	.58	(.0856)	9.72
G_1_	.83	Algin	21.4	16.2	.76	5	95.3	.87	(.0856)	4.60
G_2_	.82	CMC	21.4	14.8	.69	5	101.3	.44	.0814	(4.45)
G_2_	1.23	CMC	21.4	22.1	1.03	5	100.5	.63	.0804	(4.45)
G_2_	0.82	CMC	10.0	14.8	1.48	11	47.6	1.15	.0829	(9.65)
G_2_	.82	Algin	21.4	15.7	0.73	5	95.0	1.57	.0837	(4.60)
G_2_	1.23	Algin	21.4	23.5	1.10	3	94.5	1.71	.0831	(4.60)
M_1_	0.036	CMC	21.4	14.0	0.65	4	289.4	0.14	5.01	(4.45)
M_2_	2.50	CMC	21.4	16.7	.78	5	34.1	1.40	0.010	(4.45)

a G_1_ and G_2_ are different preparations of d-glucose-1-*t*; M_1_ and M_2_ are d-mannitol-*1-t*.

b *m*′ is the weight of the radioactive sample contained in the film of weight *m.*

c $\sqrt{\frac{\sum d^{2}}{n-1}}\times 100$

d *μ*c=cps×*m*×*k*. Values in parentheses were used as standards.

**Table 2:** Counting rate of *d*-glucose-t as a function of film thickness

d-Glucose-*t*^a^	Volume of film-forming solution	Total solids	Film thickness	Average count^b^	Standard deviation	Counting efficiency cps/dps
						
*mg*	*ml*	*mg*	*mg/cm*^2^	*cps*	%	%
0.02	0.02	0.02	0.000934	30.9	2.3	48.8
.05	.05	.05	.00234	74.3	2.9	46.9
.10	.10	.10	.00467	142.2	3.4	44.9
.20	.20	.20	.00934	232.7	5.7	36.7
.50	.50	.50	.0234	499	4.4	31.5
1.00	1.00	1.00	.0467	750	4.0	23.7
2.00	2.00	2.00	.0934	1103	3.4	17.4
1.00	1.00	c9.00	0.420	d1971	2.1	6.9
1.00	1.00	17.00	.794	2108	2.0	3.9
1.00	1.00	25.00	1.170	2108	0.6	2.7

a The activity of the d-glucose-*t* was 0.0856 *μ*c/mg. The “effective area” of the planchet was 21.4 cm^2^.

b Average of three planchets, each counted to 10,000 counts.

c In the last three experiments, the amount of d-glucose-*t* was constant, and the weight of film was increased by use of an Algin solution.

d The observed cps and the total dps in the last three experiments were multiplied by the dilution ratio, i.e., by 9,17, and 25, respectively, to reduce all results to a common specific activity.

## Footnotes

2 Figures in brackets indicate the literature references at the end of this paper.

3 A critical study of the energy loss and penetration range of *beta* particles is given in [8]. In general, the range increases with the atomic number of the material penetrated. In the film-counting method described here, the films are of organic materials, and have approximately the same elemental composition. In the presence of elements of higher atomic number, the greater depth of penetration would require a thicker film for infinite thickness, and use of a different factor for converting counts to microcuries.

4 The counting of earbon-14 from thin films of agar has been reported by McCready [10]. See also a modification of the method [11].

5 The dye serves to show the uniformity of the film.

6 Care must be used to obtain a grease-free surface on the planchet. In this laboratory the planchets are cleaned in a solution of sodium hydroxide or “Versene” immediately before use.

7 Value derived from the average of two half-thickness values experimentally determined by Dorfman.
